# Clinical validation of a novel multiplex real-time PCR for diagnosing community-acquired pneumonia in the emergency department: a promising diagnostic tool for *Mycoplasma pneumoniae*

**DOI:** 10.3389/fmicb.2026.1979614

**Published:** 2026-10-07

**Authors:** Liuyang Yang, Chaomin Guo, Qiang Zhao, Jiyong Yang, Heng Zhang, Yongzhi Zhai, Yanning Ma

**Affiliations:** 1 Department of Laboratory Medicine, The First Medical Center, Chinese PLA General Hospital, Beijing, China; 2 Department of Emergency, The First Medical Center, Chinese PLA General Hospital, Medical School of Chinese PLA, Beijing, China

**Keywords:** antimicrobial resistance genes, community-acquired pneumonia, diagnostic accuracy, metagenomic next-generation sequencing, multiplex real-time PCR, *Mycoplasma pneumoniae*

## Abstract

Community-acquired pneumonia (CAP) is a global public health burden with high morbidity, but timely diagnosis is hindered by limitations of conventional methods. We validated a novel multiplex real-time PCR (mRT-PCR) for CAP diagnosis in the emergency department, focusing on *Mycoplasma pneumoniae* (*M. pneumoniae*). A retrospective cohort of 371 CAP patients (2019–2021) from the Chinese PLA General Hospital was enrolled. The panel targeted 85 pathogens (40 bacteria, 18 viruses, 17 fungi, 10 other) plus 8 resistance genes, with a turnaround time (TAT) under 4 h and a cost of approximately US$100 per test. mRT-PCR showed performance comparable to mNGS for common pathogens (concordance >69% for bacteria/fungi) and high concordance with the final clinical diagnosis. Notably, it detected *M. pneumoniae* in 36.7% (136/371) of cases, outperforming conventional methods by identifying 204 exclusive positives (55.0% of cohort), with *M. pneumoniae* (40%) and viruses (30%) predominating. Polymicrobial infections occurred in 36.1% of cases; *M. pneumoniae* co-infections (38 cases) showed elevated neutrophils and reduced lymphocytes vs. single infections. An independent reference standard analysis excluding both index tests showed that 24.1% (210/873) of calls were independently corroborated - high for influenza, SARS-CoV-2 and *M. pneumoniae*, lower for *S. pneumoniae*, consistent with the limited sensitivity of sputum culture. Unlike conventional syndromic panels such as FilmArray, which profile fewer targets without concurrent resistance-gene detection, this mRT-PCR combines broad coverage with rapid TAT and low cost, making it promising for timely CAP diagnosis, particularly in resource-limited settings and for targeted therapy.

## 1 Introduction

Lower-respiratory-tract infections (LRTIs), encompassing community-acquired pneumonia (CAP), hospital-acquired pneumonia (HAP), and ventilator-associated pneumonia (VAP), pose a global public health burden with significant morbidity and mortality. In the United States, CAP affects approximately 248 per 10,000 adults annually (Jain et al., 2015), while the 30-day mortality rate for moderate-to-severe CAP patients admitted to intensive care units (ICUs) ranges from 23 to 47% (Wunderink and Waterer, 2017). In China, LRTIs are equally pressing: a multicenter study involving 16,585 CAP patients revealed that children aged ≤5 years (37.3%) and adults ≥65 years (28.7%) constituted the predominant patient groups, contrasting sharply with the 26–45 age group (9.2%) (Liu et al., 2013). This age distribution underscores the disease’s impact on vulnerable populations, particularly given China’s 2012 pneumonia mortality rate of 17.46 per 100,000 (Cao et al., 2018).

Diagnostic challenges in CAP stem from the limitations of conventional microbiological methods (Ramirez et al., 2020). Cultivation-based approaches for viruses, atypical pathogens (e.g., *Mycoplasma pneumoniae*, *Chlamydia pneumoniae*), and parasites face hurdles such as low sensitivity, lengthy incubation times (up to 28 days for tuberculosis), and susceptibility to contamination (Gadsby et al., 2019). These constraints have spurred the adoption of molecular diagnostics: commercial multiplex polymerase chain reaction (PCR) panels now enable simultaneous detection of 20–50 respiratory pathogens within 4–6 h, outperforming culture in speed and breadth. However, their reliance on predefined target panels may miss emerging or rare pathogens, while such panels lack the unbiased genome-wide profiling capability of metagenomic next-generation sequencing (mNGS).

In recent years, mNGS has emerged as a promising tool in the clinical diagnosis and management of CAP, as it enables the identification of both known and novel pathogens without prior target bias. A recent study revealed that mNGS detected 36.1% more cases of multibacterial infections in CAP compared with traditional culture methods, while another study highlighted its advantages in diagnosing antibiotic-resistant bacteria. However, mNGS also has certain limitations. First, it comes with a high-cost burden. Second, its turnaround time is approximately 2 days. Third, its large-scale implementation in primary and frontline hospitals remains challenging.

In this study, we aimed to establish a comprehensive microbial mRT-PCR platform for diagnosis of CAP patients. We recruited 371 CAP cases from Chinese PLA General Hospital between 2019 and 2021, and we performed the clinical validation of a novel mRT-PCR for diagnosing community-acquired pneumonia (CAP) in the emergency department.

Distinct from established syndromic multiplex panels (e.g., the FilmArray Respiratory Panel, which typically interrogates 20–50 pathogens without concurrent antimicrobial-resistance gene profiling), the present mRT-PCR platform integrates 85 pathogens and 8 resistance genes within a turnaround time of <4 h.

## 2 Methods

### 2.1 Study design and patient cohort

Between October 1, 2019, and October 31, 2021, a total of 512 patients admitted to the Emergency Department of the First Medical Center of the People’s Liberation Army General Hospital in Beijing, China underwent screening for review and enrollment in this study (Figure 1). Patients were enrolled in this retrospective study to validate the mRT-PCR assay if they had at least one of the following: (1) fever of undetermined origin (Jacoby and Swartz, 1973), (2) respiratory system symptoms, such as coughing and phlegm production, (3) radiographic abnormality. Exclusion criteria were as follows: (i) sputum specimens with a transport delay exceeding 24 h, (ii) sample collection performed >48 h after admission, and (iii) refusal to participate in the study. Demographic characteristics of the cohort are provided in Table 1. This study was approved by the institutional review boards at the First Medical Center of the General Hospital of the People’s Liberation Army (ethical approval number: S2024-795-01).

**Figure 1:**
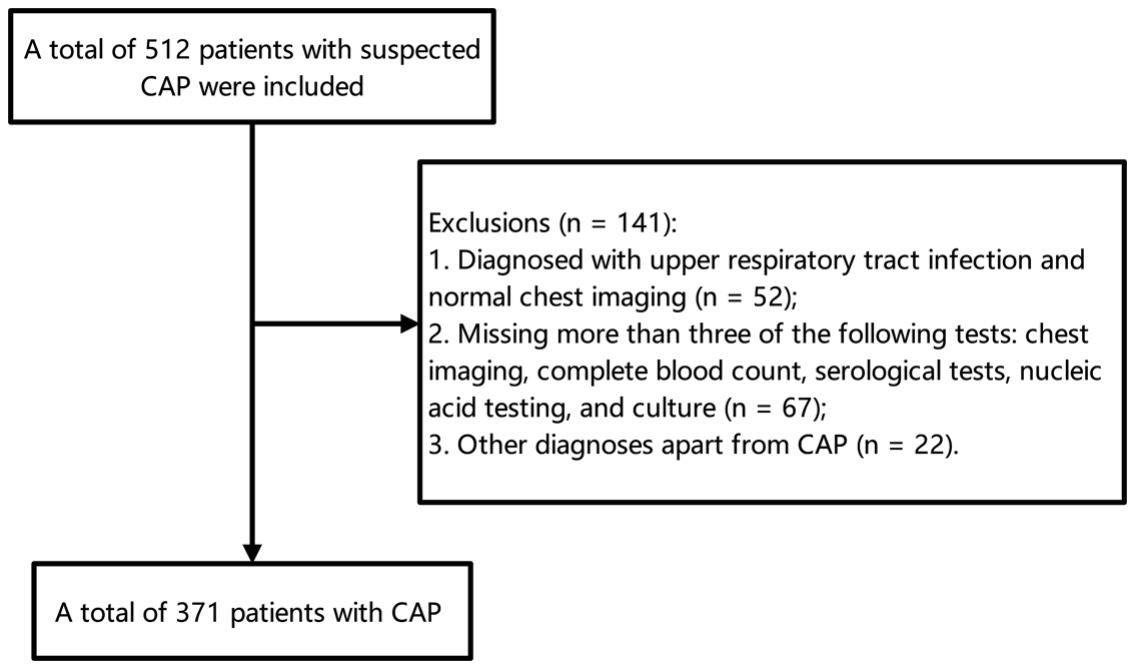
Flowchart of study participants. CAP, community-acquired pneumonia.

**Table 1:** Demographic and clinical characteristics of the cohort.

Characteristics	Retrospective study (*n* = 371)
Age (years)	
Median (IQR)	45 (30.50–64.50)
Range	14–94
Sex, *n* (%)	
Female	163 (43.9)
Male	208 (56.1)
Immunocompromised, *n* (%)	1 (0.27)
Underlying conditions, *n* (%)	
Respiratory diseases	26 (7.01)
Hematologic tumors	3 (0.81)
Blood system diseases	4 (1.08)
Solid organ tumors	7 (1.89)
Autoimmune diseases	15 (4.04)
Metabolic diseases	26 (7.01)
Circulatory system diseases	41 (11.05)
Digestive system diseases	12 (3.23)
Nervous system diseases	18 (4.85)
Urinary system diseases	12 (3.23)
Mental diseases	1 (0.27)
WBC, median (IQR) (×10^9^/L)	7.85 (6.04–10.36)
Range	1.22–28.86
Neutrophil ratio, median (IQR) (%)	0.74 (0.65–0.80)
Range	0.27–0.97
Lymphocyte ratio, median (IQR) (%)	0.17 (0.11–0.23)
Range	0.01–0.64
Monocyte ratio, median (IQR) (%)	0.08 (0.06–0.10)
Range	0.005–0.26
CRP, median (IQR) (mg/L)	3.52 (1.48–6.98)
IL-6, median (IQR) (pg/mL)	35.50 (15.86–68.15)
Pulmonary infection type	
Single pathogen pulmonary infection	237 (63.88)
Multiple microbial pulmonary infections	134 (36.12)

IQR, interquartile range; WBC, white blood cell; CRP, C-reactive protein; IL-6, interleukin-6.

### 2.2 Multiplex real-time PCR (mRT-PCR)

Nucleic acids were extracted from sputum specimens using a magnetic bead-based extraction kit (Broad-Spectrum Pathogen Kit II; Hebei Pathogeno Shengkang Medical Technology Co., Ltd.) according to the manufacturer’s instructions. The extracted nucleic acids were subsequently subjected to multiplex real-time PCR using the mRT-PCR panel, which simultaneously targets 85 pathogens and 8 antimicrobial-resistance genes (see Section 3.1 and Supplementary Figure S1). Amplification and fluorescence detection were performed on a real-time PCR instrument following the manufacturer’s recommended thermal-cycling protocol. Each run incorporated positive and negative controls as well as internal DNA and RNA extraction and amplification controls. Results were accepted only when the controls met predefined acceptance criteria (positive controls with at least 90% of targets detected at Ct ≤ 28; negative controls with no amplification or Ct ≥ 30; internal controls at Ct ≤ 25). Targets were interpreted as strong positive (Ct ≤ 20), weak positive (20 < Ct ≤ 30), gray zone (30 < Ct ≤ 32; prompting retesting), negative (no amplification with internal control Ct ≤ 28), or invalid (no amplification with internal control Ct > 28; such samples were re-extracted and re-tested). All bacterial isolates recovered from the same sputum specimens were identified by standard microbiological methods and subjected to antimicrobial susceptibility testing; minimum inhibitory concentrations were interpreted according to the Clinical and Laboratory Standards Institute (CLSI) M100 performance standards, 33rd edition (Clinical and Laboratory Standards Institute, 2023). For analytical validation, limit-of-detection and precision were established for 10 representative pathogen targets spanning the four pathogen categories covered by the panel (bacteria, viruses, fungi, and atypical bacteria; Supplementary Table S1); the remaining targets were assessed for analytical specificity through cross-reactivity and non-interference testing.

### 2.3 Metagenomic next-generation sequencing (mNGS)

Clinical samples underwent metaDNA-seq and/or metaRNA-seq per established protocols (Zhang et al., 2024). DNA/RNA were extracted using QIAamp® kits (Qiagen) (Amar et al., 2021). For metaRNA-seq, total RNA was rRNA-depleted (Ribo-Zero, Illumina) and reverse-transcribed to cDNA (Thermo Fisher). Libraries were prepared using Nextera XT kits (Illumina) (Miller et al., 2019), pooled, and sequenced on Illumina NextseqCN500 (75-cycle SE, ~20 M reads/library). Negative controls included healthy donor PBMCs (10^5^ cells/mL) and sterile water (NTC) (Li et al., 2018). Reads were trimmed by Trimmomatic (Bolger et al., 2014), low-complexity sequences removed by Kcomplexity, and human reads filtered via BWA-mapping to hg38 (Li and Durbin, 2009). Reference databases included hg38 and 35,737 microbial genomes (NCBI), built with Kraken2 v2.0.8beta. Taxonomic classification used Kraken2 (Wood and Salzberg, 2014), with species-level quantification via Bracken (Kobus et al., 2020). Positive detection required RPM ratio ≥10 (sample/NTC) for taxa with background reads, or RPM ≥ 0.05 for clean backgrounds, with 5%/10% penalties for species/genus assignments (Gu et al., 2020).

### 2.4 Clinical diagnoses by clinician committees

The clinician committees, consisting of specialists in respiratory medicine, clinical laboratory physicians, and radiologists, utilized the diagnostic standards for CAP (Metlay et al., 2019). These diagnoses drew on clinical manifestations, findings from lung imaging, outcomes of routine tests (such as culture, PCR, and lung imaging), as well as results from mRT-PCR or mNGS. Because no single microbiological test provides a perfect gold standard for CAP pathogen identification, the integrated committee diagnosis was adopted as a composite reference standard (CRS) against which the performance of mRT-PCR (and mNGS) was evaluated.

### 2.5 Independent reference standard for sensitivity analysis

Because the composite reference standard (CRS) described in section 2.4 incorporated mRT-PCR and mNGS results, it is not fully independent of the index tests and may yield optimistic performance estimates. To examine this circularity, we additionally constructed an independent reference standard (IRS) that excluded both molecular assays. Accuracy estimates were restricted to the 15 targets for which an independent comparator exists (components i–iii below); the remaining targets have no routine comparator at our institution, were adjudicated clinically (component iv), are reported separately (Part B of Supplementary Table S3), and were not used to compute the accuracy estimates. For each target detected by mRT-PCR, the IRS was defined as: (i) bacteria and fungi—conventional sputum culture on blood and chocolate agar; (ii) *Mycoplasma pneumoniae*—serum IgM by particle agglutination; (iii) influenza A/B and SARS-CoV-2—routine single-plex real-time PCR or antigen testing performed in the clinical microbiology laboratory; (iv) all remaining targets for which no routine comparator was available at our institution—an independent physician-panel adjudication incorporating the mRT-PCR cycle-threshold (Ct) value, chest imaging, and the clinical course. A mRT-PCR positive call was counted as independently corroborated only when the identical target was identified by one of these IRS components. Clinicians who formed the CRS were not blinded to the mRT-PCR and mNGS results; the IRS analysis was therefore performed to provide an assessment that is independent of the index tests.

### 2.6 Statistical analysis

Statistical evaluations were conducted with SPSS 25.0 software. Agreement between mRT-PCR and mNGS was quantified with Cohen’s kappa and its 95% confidence interval, and discordant calls were compared with McNemar’s test; categorical comparisons of detection rates used the chi-square or Fisher’s exact test as appropriate. A two-sided α of 0.05 was considered significant. Given the descriptive aim of the study, no formal *a priori* sample-size computation was carried out and no correction for multiple comparisons was applied; missing data were handled by the exclusion rules stated in section 3.2 (patients lacking more than three of the core tests were excluded).

## 3 Results

### 3.1 The novel multiplex real-time PCR (mRT-PCR) design

The panel was designed based on an extensive review of source materials, including clinical expert consensus statements and infection-related literature. This led to the development of a novel multiplex real-time PCR (mRT-PCR) assay, which targets 85 pathogens and 8 resistance genes—encompassing 40 bacteria, 18 viruses, 17 fungi, 3 mycoplasmas, 3 chlamydia, and 4 other atypical pathogens (as illustrated in Supplementary Figure S1 and Figure 2). A complete list of the target species identified by this mRT-PCR panel is provided in Supplementary Figure S1, while the detection workflow of the assay is depicted in Figure 2. The turnaround time (TAT) for the mRT-PCR assay was within 4 h. At a cost of <US$100 per test, this mRT-PCR assay was more economical than both mNGS and tNGS (Table 2).

**Figure 2:**
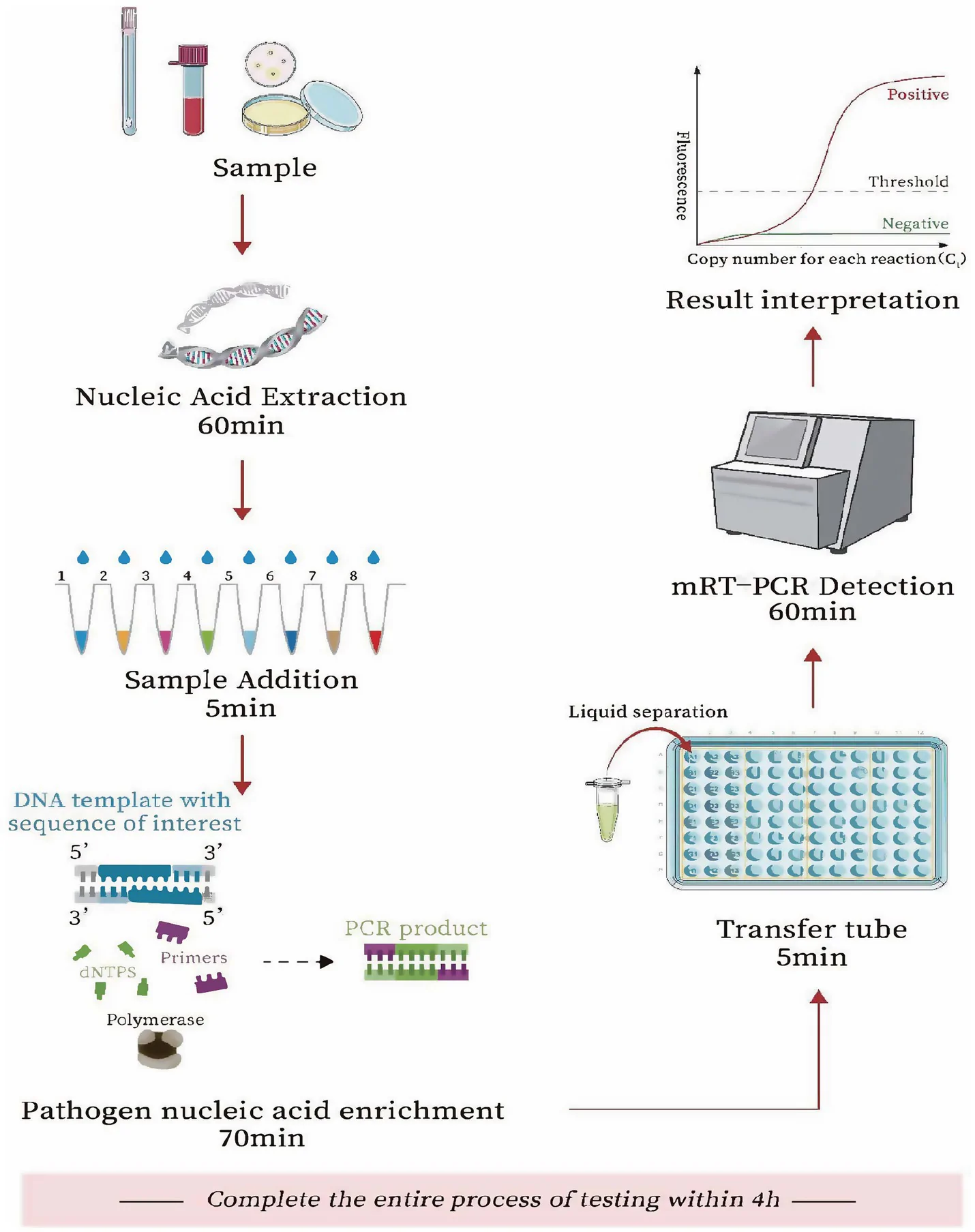
The mRT-PCR detection workflow (4 h turnaround). From nucleic acid extraction through multiplex real-time PCR amplification to automated result interpretation.

**Table 2:** Technical and practical comparison between mRT-PCR platform, mNGS and tNGS.

Event	mRT-PCR platform (this study)	Metagenomic NGS (mNGS)	Target NGS (tNGS)
Target spectrum	85 pathogens, 8 drug resistance genes	>20,000 species	>3,000 species
Pathogen coverage (sputum)	85 pathogens	All covered	Almost all covered
Time for the identification of pathogens	Nearly 4 h	16–24 h	16–23 h
Cost	∼100 US dollars (USD).	DNA procedure: ∼500 USD. DNA + RNA procedure: ∼900 USD.	∼300 USD.
Advantages	1. Rapid and efficient, capable of simultaneously detecting specific pathogens and drug resistance genes. 2. Relatively low cost, suitable for detection needs with clear targets. 3. Accurate quantification and relatively simple result interpretation.	1. The widest coverage, capable of detecting all types of pathogens, including novel or rare pathogens. 2. Culture-independent, directly applicable to clinical samples, and unbiased detection. 3. Potential for antibiotic/antiviral resistance prediction and functional analysis.	1. High-throughput, using hybridization probe capture to enrich target sequences, sensitive in detecting pathogens within specific spectra. 2. Suitable for low-biomass or high-host-contamination samples, with excellent performance in detecting fungi, mycobacteria, etc. 3. Moderate sequencing data volume (20 M), balancing detection efficiency and accuracy.
Drawbacks	1. Limited coverage, only targeting the preset 85 pathogens and drug resistance genes. 2. Subject to amplification bias, difficult to distinguish between live and dead cells. 3. High risk of missing rare pathogens or those not included in the detection spectrum.	1. The highest cost, with DNA + RNA detection costing nearly 1,000 USD. 2. Laborious and complex operational procedures, prone to contamination. 3. Difficult to distinguish between live cells, dead cells, and active cells, requiring profound microbiological expertise for result interpretation. 4. DNA and RNA detection need to be operated separately, taking a long time.	1. Still interfered by host genetic background and microorganisms with high genomic similarity. 2. Weak in detecting common bacteria such as *Pseudomonas aeruginosa* and *Klebsiella pneumoniae*. 3. Dependent on databases of known pathogens, with limited potential for discovering novel pathogens, and certain amplification bias exists.

Additionally, the limit of detection (LoD) was measured for ten representative pathogens (*S. pneumoniae, S. aureus, K. pneumoniae, H. influenzae, C. albicans, A. fumigatus, M. pneumoniae*, Cytomegalovirus [CMV], Influenza A virus [FluA], Influenza B virus [FluB]). The lower limit of detection was 1,000 copies/mL for the four bacteria (*S. pneumoniae*, *S. aureus*, *K. pneumoniae*, *H. influenzae*), 2,000 copies/mL for *Candida albicans*, 4,000 copies/mL for *Aspergillus fumigatus*, 500 copies/mL for *M. pneumoniae*, and 500 copies/mL for the three viruses (CMV, FluA, FluB) (Supplementary Table S1). Both within-run precision and between-run precision were <2.0%. These ten representative pathogens were assessed using value-assigned nucleic-acid reference materials obtained from the BeNa Culture Collection (BNCC; Henan Engineering Research Center of Industrial Microorganisms, Henan, China). These are nucleic-acid standards rather than live bacterial or viral cultures; each is a non-viable preparation quantified by fluorescence-based and droplet digital PCR, and is traceable to the parent strain or culture-collection accession certified in the supplier’s certificate of analysis. The catalog numbers and traceability of all materials are listed in Supplementary Table S1.

### 3.2 Patient characteristics

From a cohort of 512 patients admitted to the Emergency Department with suspected community-acquired pneumonia (CAP), we identified 371 patients with retrospectively confirmed CAP (Figure 1). A total of 141 patients were excluded, including 52 diagnosed with upper respiratory tract infection and having normal chest imaging; 67 with missing data for more than three of the following tests (chest imaging, complete blood count, serological tests, nucleic acid testing, and culture); and 22 with diagnoses other than CAP. Among these 371 patients, the median age was 45 years, and 56.1% were male (Table 1). White blood cell counts (WBC, 7.85 × 10^9^/L), neutrophil percentage (74%), C-reactive protein (CRP, 3.52 mg/L), and interleukin-6 (IL-6, 35.50 pg./mL) were all elevated above the upper limits of normal reference ranges (Table 1). These laboratory findings were consistent with the diagnostic criteria for CAP.

### 3.3 Evaluation of clinical diagnostic efficacy of mRT-PCR and mNGS methods

To assess the clinical diagnostic efficacy of mRT-PCR and mNGS in patients with community-acquired pneumonia (CAP), we analyzed 30 samples (Figure 3). For bacterial pathogens, *Haemophilus influenzae* (*H. influenzae*), *Moraxella catarrhalis*, *Pseudomonas aeruginosa* (*P. aeruginosa*), *Acinetobacter baumannii*, and *Klebsiella pneumoniae* demonstrated nearly uniform co-detection by both methods. In contrast, *Staphylococcus aureus* (*S. aureus*) and *Streptococcus pneumoniae* (*S. pneumoniae*) showed concordance rates of 75 and 76.5%, respectively. For *Mycoplasma pneumoniae* (*M. pneumoniae*), 69.2% (9/13) of positive samples were detected by both techniques, with 15.4% (2/13) identified exclusively by mRT-PCR and another 15.4% (2/13) exclusively by mNGS. In fungal detection, *Aspergillus flavus*, *Candida lusitaniae*, and *Aspergillus fumigatus* were consistently detected by both methods. *Chlamydia psittaci*, an atypical bacterial pathogen, was also consistently detected by both methods. At the pathogen level, the overall agreement between mRT-PCR and mNGS was substantial (kappa = 0.538, 95% CI 0.425–0.651); at the patient level, both platforms detected at least one of the committee-adjudicated pathogens in 28 of 30 paired samples (93.3%, 95% CI 78.7–98.2); in the two discordant samples the committee-adjudicated pathogen was recovered by mRT-PCR but not reported by mNGS. For *Candida albicans*, 83.3% (10/12) of positive samples were co-detected, with 16.7% (2/12) identified exclusively by mRT-PCR and no samples detected solely by mNGS. Collectively, these results indicate that mRT-PCR exhibits similar clinical diagnostic efficacy to mNGS. Against conventional sputum culture in the same 30-paired-sample subset, the two assays recovered culture-positive isolates at an identical rate—69.6% (16 of 23 isolates) for both mRT-PCR and mNGS—and did not differ significantly (McNemar exact test, *p* = 1.000); five isolates (21.7%) were recovered by neither platform. Taking the individual culture isolate as the unit of analysis, without correction for within-patient clustering, these data indicate that the two platforms perform equivalently against this comparator and that mRT-PCR is not specifically disadvantaged; the residual non-recovery reflects the recognized imperfect correspondence between sputum culture and nucleic-acid-based detection rather than a defect of either assay.

**Figure 3:**
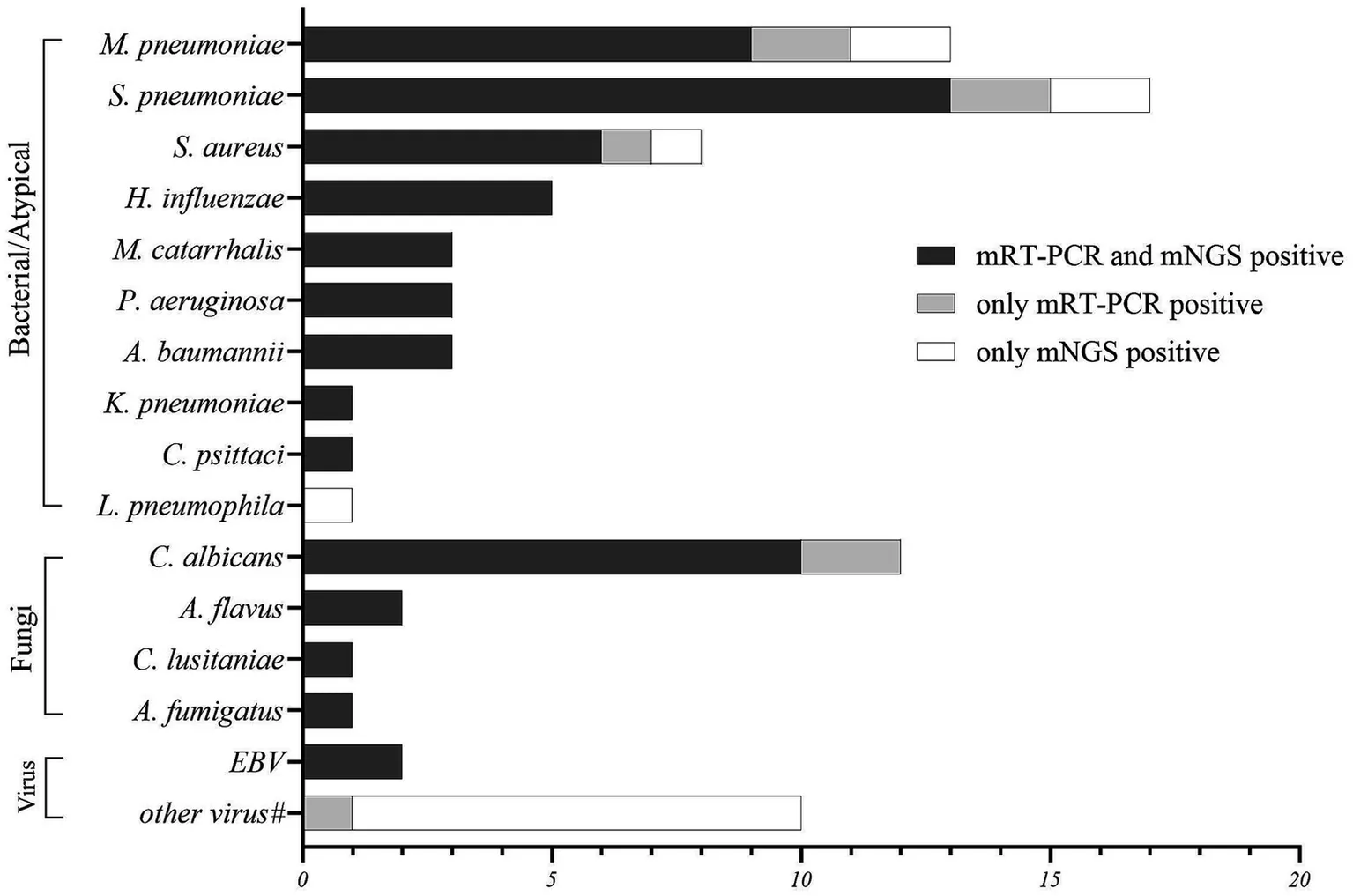
Evaluation of clinical diagnostic efficacy of mRT-PCR and mNGS methods. The horizontal axis represents the number of detected pathogens, and the vertical axis represents the pathogen names. The black box represents detection of both mRT-PCR and mNGS, the gray box represents detection of only mRT-PCR, and the white box represents detection of only mNGS.

### 3.4 Independent reference standard sensitivity analysis

To quantify the circularity concern raised during review, we audited all 873 positive mRT-PCR calls against the IRS (section 2.5). Overall, 210 calls (24.1%) were independently corroborated by culture, serology, or routine PCR, and a further 6 (0.7%) were consistent only with the free-text clinical diagnosis; the remaining 657 calls (75.3%) lacked any independent corroboration, concentrated among targets for which our hospital performs no routine comparator test (see below). Target-level diagnostic accuracy for every target with an available routine comparator, together with the corresponding 2×2 tables, is provided in Supplementary Table S3. Pooled across the 15 target-specific comparisons against the IRS in the full 371-patient cohort, mRT-PCR sensitivity was 70.2% (95% CI 64.8–75.1), specificity 92.0% (95% CI 91.3–92.7), with a positive likelihood ratio of 8.83 and a negative likelihood ratio of 0.32 (Supplementary Table S3).

*Streptococcus pneumoniae* was detected 125 times but culture-confirmed in only 9 (7.2%). This low corroboration reflects the well-recognized insensitivity of sputum culture on blood and chocolate agar for *S. pneumoniae*—especially after antibiotic exposure (Yang et al., 2005; Yoshii et al., 2016)—rather than mRT-PCR false positivity; molecular assays routinely recover *S. pneumoniae* that culture misses. Of note, 71 patients whose sputum yielded only normal flora were mRT-PCR-positive for *S. pneumoniae*; these detections were retained only when supported by Ct value, imaging, and clinical course, acknowledging that sputum carriage cannot be fully excluded in every case. Target-level corroboration for *S. pneumoniae* and all other targets is detailed in Supplementary Table S3.

Epstein–Barr virus, human rhinovirus, respiratory syncytial virus, human metapneumovirus, cytomegalovirus, herpes simplex virus 1, and *Chlamydia psittaci* accounted for 181 positive calls with 0% independent corroboration. This is because our institution offers no routine diagnostic test for these agents in respiratory specimens; they were clinically adjudicated by the physician panel using Ct value, imaging, and history. The absence of a comparator therefore reflects a gap in routine diagnostics, not evidence of false positivity.

Consistent with the limited precision of the 30-sample head-to-head comparison (section 3.3), a post-hoc power calculation indicated that *n* = 30 provides only 49.1% power to detect a true kappa of 0.90 between two methods; 80% power would require 63–393 samples. These 30 patients constituted a non-random, convenience subset who concurrently underwent mNGS (typically for severe or diagnostically uncertain cases), so the comparison is also subject to selection bias, as acknowledged in the limitations. The IRS audit across the 371-patient cohort therefore provides the more robust assessment of mRT-PCR performance, and the agreement statistics reported in section 3.3 should be interpreted as low-precision estimates.

### 3.5 Microbiological detections

In this study, all final infecting pathogens in patients were identified by a committee of clinicians. Among the 371 patients, 237 cases were caused by a single pathogen, followed by 127 cases involving two pathogens and 7 cases involving three pathogens (Figure 4A). Polymicrobial infections were detected in 36.1% (134/371) of cases via mRT-PCR testing. Here, polymicrobial infection was defined as the detection of two or more pathogens in a single patient.

**Figure 4:**
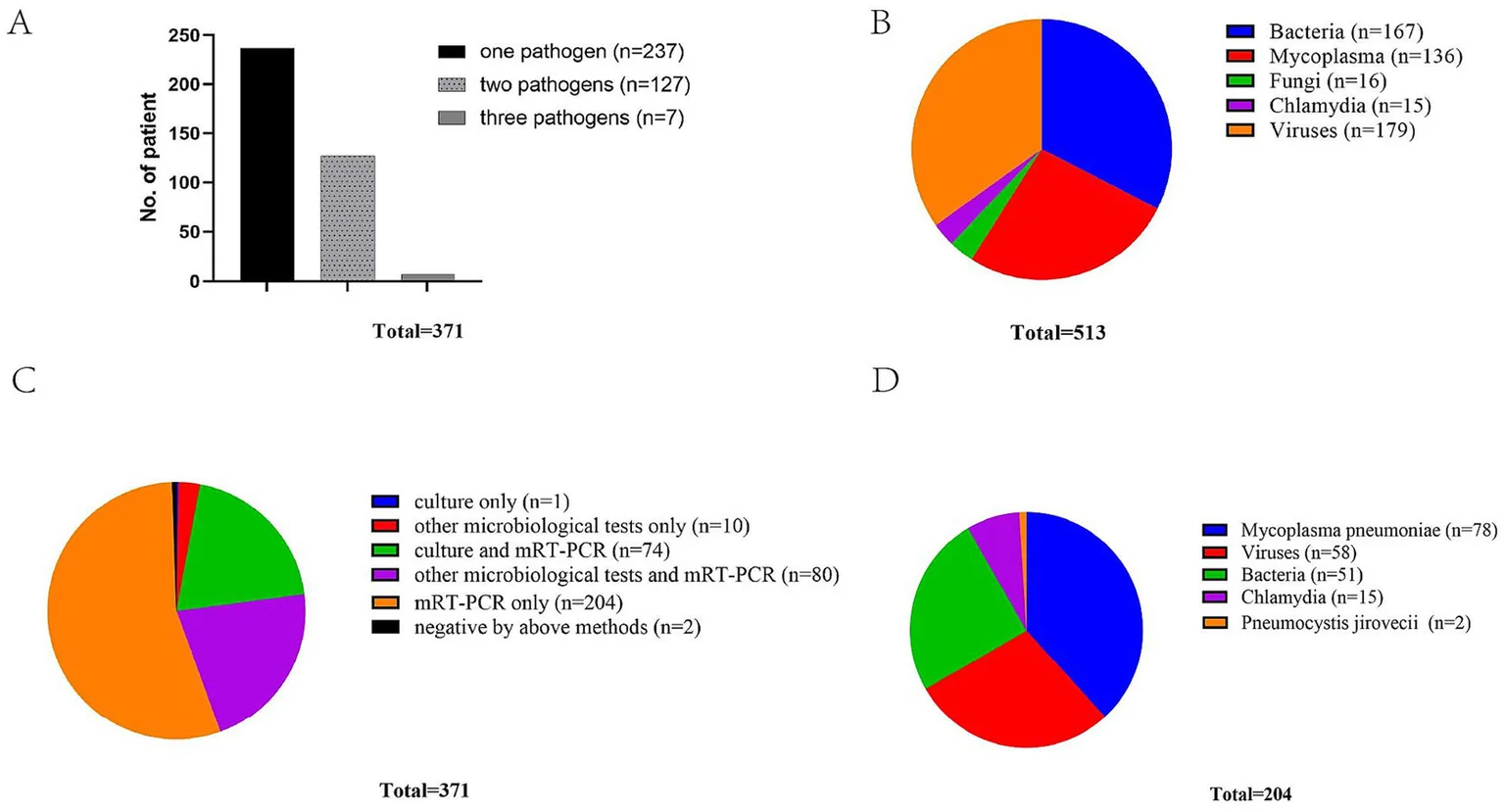
Clinical usability of mRT-PCR for CAP pathogen identification. **(A)** The bar chart represents the number of patients infected with one pathogen (*n* = 237), two pathogens (*n* = 127), and three pathogens (*n* = 7). **(B)** Pie chart showing the proportion of pathogens identified as causal pathogens by clinical doctor in this study (*n* = 513). **(C)** Pie chart showing the proportion of patients (total 371 patients) diagnosed by the different methods, including 10 diagnosed solely by CMTs and 2 solely by mNGS. **(D)** Pie chart showing the proportion of patients diagnosed only by mRT-PCR method (*n* = 204).

Across the entire cohort, a total of 513 pathogens were detected, comprising 179 viruses, 167 bacteria, 136 mycoplasmas, 16 fungi, and 15 chlamydiae (Figure 4B). The viruses with the highest detection rates were SARS-CoV-2 (46 cases) and influenza A virus (39 cases). Among the 167 bacterial pathogens, 120 were Gram-negative, with the top three being *H. influenzae* (*n* = 66), *Klebsiella pneumoniae* (*n* = 18), and *P. aeruginosa* (*n* = 12). In addition, 47 were Gram-positive or acid-fast organisms, including *S. pneumoniae* (*n* = 36), *S. aureus* (*n* = 6), *M. tuberculosis* (*n* = 1; an acid-fast bacillus rather than a true Gram-positive organism), and other Gram-positive species. As shown in Figure 4B, 36.67% (136/371) of cases tested positive for *M. pneumoniae* infection (a rate distinct from the 36.1% polymicrobial infection frequency reported above), indicating that mRT-PCR exhibits significant advantages in detecting *M. pneumoniae*. Additionally, among the 16 fungal strains detected, the most common were *Candida albicans* (*n* = 10), Aspergillus flavus (*n* = 2), and Pneumocystis jirovecii (*n* = 2).

Among the eight targeted resistance genes, MecA, VIM, KPC, NDM, and IMP were detected in the clinical strains, with their corresponding antibiotic resistance rates summarized in Supplementary Table S2; these resistance phenotypes were interpreted in accordance with the CLSI M100 performance standards, 33rd edition (Schuetz et al., 2025). AmpC, MCR-1, and VanA were not detected in this study.

### 3.6 Clinical usability of mRT-PCR for CAP pathogen identification

We evaluated the detection performance of mRT-PCR, culture methods, and other conventional testing methods (CMTs) in a retrospective study involving 371 patients with CAP. mRT-PCR returned positive results in 358 of 371 patients; among these, 204 (55.0% of the cohort) were exclusively detected by mRT-PCR. Figure 4D summarizes all pathogens uniquely identified by mRT-PCR: *M. pneumoniae* and viruses constituted approximately 40 and 30% of these microbes, respectively. The most frequently detected viruses by mRT-PCR alone were SARS-CoV-2 (*n* = 15), *Influenza A virus* (*n* = 11), and Adenovirus (*n* = 10), while *H. influenzae* (*n* = 30) and *S. pneumoniae* (*n* = 11) were the predominant bacteria. These findings highlight mRT-PCR’s significant advantages in detecting *M. pneumoniae*, SARS-CoV-2, *Influenza A virus*, *H. influenzae*, and *S. pneumoniae* in CAP.

As shown in Figure 4C, 10 patients were solely diagnosed by CMTs, primarily via antibody detection (9 cases) and nucleic acid testing (1 case). This discrepancy may reflect temporal delays in immune responses, where antibody positivity persisted despite negative nucleic acid results. Notably, one patient infected with *Acinetobacter pittii* was identified only by culture, likely due to the absence of target sequences in mRT-PCR panels. The remaining two negative cases were ultimately diagnosed by mNGS.

In this study, mRT-PCR showed a great advantage in detecting *M. pneumoniae* in CAP (Figure 4D). As shown in Table 3, patients with *Mycoplasma pneumoniae* pneumonia (MPP) were divided into the *M. pneumoniae* infection group (MPP group; *n* = 98) and the *M. pneumoniae* co-infection group (MPP co-infection group; *n* = 38). The MPP co-infection group comprised 24 bacteria and 14 viruses. We compared the levels of WBC, neutrophils, lymphocytes, monocytes, CRP, and IL-6 between the two groups, and compared the pulmonary CT scan results between these two groups (Table 3). The MPP co-infection group showed significantly higher levels of neutrophils (*p* = 0.004) and lower levels of lymphocytes (*p* = 0.003) compared with the MPP group. For the pulmonary CT scan results, both the MPP group and the MPP co-infection group had a higher rate of right lung infection (39.80 and 42.11%, respectively).

**Table 3:** Clinical usability of mRT-PCR for CAP pathogen identification for *Mycoplasma pneumoniae*.

Characteristics	MPP group	MPP co-infection group	*p* value
No. of the patients (*n*)	98	38	
Combined pathogens	–	Bacterial (24); Virus (14)	
Inspection indicators			
White Blood Cell	6.94 (3.43, 15.30)	7.83 (3.42, 16.21)	0.072
Neutrophils	0.69 (0.27, 0.88)	0.74 (0.52, 0.92)	0.004
Lymphocyte	0.20 (0.07, 0.64)	0.15 (0.05, 0.34)	0.003
Monocyte	0.08 (0.04, 0.16)	0.08 (0.03, 0.17)	0.246
CRP	4.27 (0.09, 18.88)	3.46 (0.10, 19.73)	0.572
IL-6	28.56 (1.50, 148.50)	25.65 (1.50, 216.40)	0.946
Pulmonary CT scan			
Left lung infection	25 (25.51%)	8 (21.05%)	
Right lung infection	39 (39.80%)	16 (42.11%)	
Bilateral pulmonary infection	34 (34.69%)	14 (36.84%)	

MPP group: *Mycoplasma pneumoniae* infection; MPP co-infection group: *Mycoplasma pneumoniae* infection combined with other pathogens.

## 4 Discussion

In this study, we designed a novel mRT-PCR platform, which covered 85 pathogens and 8 resistance genes. This platform provides the broadest microbial coverage (40 bacteria, 18 viruses, 17 fungi, 3 Mycoplasma, 3 Chlamydia, and 4 other atypical pathogens) among published CAP PCR panels in China to our knowledge. This mRT-PCR platform demonstrated substantial clinical utility in detecting pathogens of community-acquired pneumonia (CAP), aligning with previous studies highlighting its superiority in identifying non-culturable microbes (Krause et al., 2014; Sugita et al., 2021; Tseng et al., 2022; Yin et al., 2024). Our findings underscore three key advantages: rapid turnaround time (within 4 h), broad coverage of 85 pathogens, and cost-effectiveness. These features address critical limitations of conventional diagnostics and mNGS, such as delayed results from culture-based assays (2–7 days) and high costs of mNGS (Sundelin et al., 2023).

Whereas commercial multiplex PCR systems such as FilmArray or Seegene have streamlined syndromic testing, they generally cover 20–50 targets and omit simultaneous resistance-gene detection. Our assay is differentiated in three respects: (i) concurrent profiling of 85 pathogens and 8 resistance genes; (ii) a sub-4-h turnaround that rivals conventional panels while remaining far faster than mNGS (≈2 days); and (iii) direct, same-cohort comparison against mNGS, which supplies comparative evidence absent from single-platform studies.

Notably, mRT-PCR showed comparable diagnostic efficacy to mNGS for bacterial (e.g., *H. influenzae, S. pneumoniae*) and fungal pathogens (*A. flavus, C. albicans*), with concordance rates exceeding 69.23% (Figure 3). The workflow differences between techniques influenced performance: mRT-PCR omits human genome depletion and enables simultaneous DNA/RNA detection, reducing costs by 80–89% compared to mNGS (Table 2). This cost–benefit makes mRT-PCR preferable for initial screening in non-critical lower respiratory tract infections, while mNGS may serve as a second-line tool for complex cases (Krause et al., 2014; Han et al., 2019). However, mRT-PCR’s targeted design missed *Acinetobacter pittii* (detected only by culture and outside the panel’s predefined target list), an inherent constraint of any targeted assay rather than a performance failure, highlighting the need for panel optimization to include emerging pathogens (Pillay et al., 2016).

In this study, clinician committees were tasked with determining the diagnoses. Given the substantial burden of severe complications among emergency department patients (Table 1), these committees were pivotal in ensuring accurate clinical diagnoses. Numerous healthcare facilities and clinics have established committees of clinicians, cross-disciplinary teams, or panels focused on clinical microbial sequencing to assist in clinical diagnosis (Han et al., 2019; Myall et al., 2021). This practice underscores the importance of deriving diagnostic conclusions by integrating clinical symptoms, lung imaging results, conventional testing approaches, and mRT-PCR outcomes.

The cohort revealed a high prevalence of polymicrobial infections (36.1%), with viruses (179/513) and mycoplasma (136/513) constituting major pathogens. This finding challenges the traditional focus on bacterial CAP, emphasizing the need for comprehensive multi-pathogen testing. *SARS-CoV-2* (46 cases) and Influenza A (39 cases) were the most frequent viruses, while *H. influenzae* (66 cases) and *K. pneumoniae* (18 cases) dominated bacterial infections. The high detection rate of *M. pneumoniae* (36.67%) aligns with its role as a leading cause of atypical pneumonia in adults (Gao and Sun, 2024). Critically, mRT-PCR identified 55.0% of cases exclusively, with *M. pneumoniae* and viruses (e.g., SARS-CoV-2, influenza A) comprising 70% of these exclusively detected microbes. This highlights mRT-PCR’s unique value in detecting organisms that evade culture or serology, such as non-culturable mycoplasma and viruses with transient viremia.

PCR-based assays are widely regarded as the gold standard for detection of *M. pneumoniae* owing to their high sensitivity (90–95%) and specificity (>99%) (Gao and Sun, 2024). Our data showed that mRT-PCR detected 136 cases of *M. pneumoniae*, including 38 co-infections with bacteria or viruses. Co-infected patients exhibited a higher neutrophil percentage and a lower lymphocyte percentage than those with *M. pneumoniae* monoinfection (Table 3). This opposing shift—neutrophil predominance together with lymphopenia—is directionally consistent with an elevated neutrophil-to-lymphocyte ratio (NLR) and reflects a heavier innate inflammatory load in co-infection. Mechanistically, the neutrophilic shift is most plausibly driven by the bacterial component of co-infections (24 of 38 cases): bacterial pathogen-associated molecular patterns (e.g., LPS, peptidoglycan) are potent inducers of neutrophil mobilization, whereas *M. pneumoniae* itself—an atypical intracellular pathogen—typically elicits a lymphocytic/monocytic response (Terpos et al., 2020). The reciprocal fall in lymphocyte percentage aligns with virus-associated lymphopenia described in respiratory viral co-infections (Yu et al., 2024) and with the immunosuppressive milieu of severe pneumonia. A raised NLR is a recognized marker of CAP severity and adverse outcomes (Ge et al., 2019; Alzoubi and Khanfar, 2021; Meng et al., 2023), supporting that MPP co-infection carries greater inflammatory burden than monoinfection. Pulmonary CT scans showed comparable right-lung involvement between the two groups, suggesting similar radiological distribution despite the differing leukocyte profile. These findings reinforce mRT-PCR’s value for early *M. pneumoniae* diagnosis, particularly in mixed infections where serology may be delayed or confounded (Zhang et al., 2011; Ding et al., 2024).

The non-random patient selection and sample size (371 cases) may introduce bias, though clinician committees minimized diagnostic errors. Sputum sampling after antibiotic therapy potentially reduced culture sensitivity for bacteria/fungi, while mNGS remained unaffected, explaining its higher sensitivity in two cases. Future studies should prospectively enroll patients before antimicrobial treatment in larger cohorts. Additionally, expanding the mRT-PCR panel to include emerging pathogens (e.g., *Acinetobacter pittii*) and more resistance genes could enhance clinical guidance for antibiotic stewardship.

This study still had some limitations. First, although the mRT-PCR platform covered the 85 microbial targets, some species (e.g., Salmonella, Enterococcus, Chlamydia urealyticum) were rarely identified as CAP pathogens; the assay also performed well on other specimen types (synovial fluid, blood, BALF; data not published) and could be extended accordingly. Second, the head-to-head comparison with mNGS was restricted to a subset of 30 paired samples, and the single-center, retrospective design may limit generalizability; prospective multi-center validation is warranted. Third, all specimens were sputum. Although clinician adjudication using Ct value, imaging and clinical course was applied, *S. pneumoniae* detected by mRT-PCR in sputum may represent colonization rather than invasion in a subset of cases, and a formal quantitative threshold for pathogen significance was not applied; bacterial findings should therefore be interpreted together with the clinical context rather than in isolation. Fourth, the cohort size of 371, although adequate for overall concordance and major pathogen groups, may be underpowered for rare targets and low-prevalence resistance genes; subgroup estimates for such targets should be interpreted with caution. Fifth, the primary performance estimates were computed against the composite reference standard (CRS), which incorporated the index tests and is therefore susceptible to circularity. The IRS sensitivity analysis (section 3.4) shows that independent corroboration is high for influenza, SARS-CoV-2, *M. pneumoniae* and culture-positive bacteria, but lower for *S. pneumoniae* (culture insensitivity) and absent for agents lacking a routine comparator; directionally, mRT-PCR performance remains favorable, yet target-specific sensitivities against a fully independent standard should be interpreted with this caveat. Sixth, the 30-sample mRT-PCR versus mNGS comparison was underpowered (post-hoc power 49.1% at a true kappa of 0.90); the observed pathogen-level kappa of 0.538 (95% CI 0.425–0.651) is a low-precision estimate, and the two molecular platforms should not be ranked on this subset alone. Seventh, we did not stratify co-infections by bacterial versus viral etiology, nor compute formal NLR from absolute leukocyte counts; subtyping co-infections and validating NLR as a severity surrogate warrant larger cohorts.

This study establishes mRT-PCR as a rapid, cost-effective, and comprehensive tool for CAP pathogen detection, particularly for *M. pneumoniae*, viruses, and antibiotic-resistant bacteria. Its performance rivals mNGS for common pathogens while offering logistical advantages for routine clinical use. Integrating mRT-PCR with clinical assessment and imaging can improve diagnostic accuracy and treatment outcomes in CAP, especially in resource-limited settings.

## Data Availability

The original contributions presented in the study are included in the article/Supplementary material. Further inquiries can be directed to the corresponding authors. De-identified participant-level datasets supporting the conclusions of this article will be made available by the authors on reasonable request, subject to approval by the Institutional Review Board of the First Medical Center of the Chinese PLA General Hospital.

## Supplementary material

SUPPLEMENTARY FIGURE S1 The mRT-PCR panel design. The panel targets 85 pathogens (40 bacteria, 18 viruses, 17 fungi, 3 Mycoplasma, 3 Chlamydia, and 4 other atypical pathogens) plus 8 resistance genes.

Supplementary Table S1 Limit of detection and inter-assay precision of the mRT-PCR panel, with the value-assigned nucleic-acid reference materials and their catalog numbers and traceability.

Supplementary Table S2 Resistance genes detected by mRT-PCR and the corresponding antibiotic resistance rates of the clinical isolates.

Supplementary Table S3 Target-level corroboration of mRT-PCR detections against the independent reference standard (IRS). Part A, the 15 targets with an available routine comparator, including the corresponding 2×2 tables and the pooled estimates; Part B, the 20 targets without a routine comparator, reported separately.
