# Supplementary material for: Clinical validation of a novel multiplex real-time PCR for diagnosing community-acquired pneumonia in the emergency department: a promising diagnostic tool for Mycoplasma pneumoniae
Source: Front Microbiol. 2026 Oct 7;17:1979614. doi: 10.3389/fmicb.2026.1979614 (PMC13683612; doi:10.3389/fmicb.2026.1979614)
Supplement: Supplementary file 1 [file Table_1.DOCX]

Table S1. Limit of detection and inter-assay precision of pathogens

| Pathogens | Sample concentration (copies/mL) | Within-run precision (%) | Between-run precision (%) | Reference material (catalog no.; traceability) |
| --- | --- | --- | --- | --- |
| S. pneumoniae | 1000 | 1.49 | 1.18 | BNCC 371909 (CMCC(B) 31001) |
| S. aureus | 1000 | 1.57 | 1.29 | BNCC 374293 (CICC 21676) |
| K. pneumoniae | 1000 | 1.66 | 1.34 | BNCC 363155 (CMCC(B) 46117) |
| H. influenzae | 1000 | 1.59 | 1.27 | BNCC 363475 (ATCC 9008) |
| C. albicans | 2000 | 1.18 | 1.11 | BNCC 365271 (ATCC 90028) |
| A. fumigatus | 4000 | 1.47 | 1.13 | BNCC 363592 (ATCC MYA-3627) |
| M. pneumoniae | 500 | 1.17 | 1.19 | BNCC 369989 (ATCC 15531) |
| Cytomegalovirus | 500 | 1.41 | 1.20 | BNCC 372530 (ATCC VR-1780) |
| Influenza A virus | 500 | 1.44 | 1.30 | BNCC 377898 (H1N1, ATCC VR-1469) |
| Influenza B virus | 500 | 1.30 | 1.23 | BNCC 373545 (BV, ATCC VR-1931) |

All materials used for LoD and inter-assay precision testing were value-assigned nucleic-acid reference materials obtained from the BeNa Culture Collection (BNCC; Henan Engineering Research Center of Industrial Microorganisms, Henan, China). These are nucleic-acid standards, not live bacterial or viral cultures; each is a non-viable preparation whose concentration was assigned by the supplier using fluorescence-based quantitative PCR and droplet digital PCR, and each is traceable to the parent strain or culture-collection accession indicated in parentheses, as certified in the supplier's certificate of analysis. ATCC, American Type Culture Collection (USA); CMCC(B), National Center for Medical Culture Collections (China); CICC, China Center of Industrial Culture Collection (China).

Cross-reactivity and non-interference: targets not subjected to LoD/precision testing were evaluated for analytical specificity; no cross-amplification among panel targets and no interference from background respiratory flora was observed.
