# Supplementary material for: Clinical validation of a novel multiplex real-time PCR for diagnosing community-acquired pneumonia in the emergency department: a promising diagnostic tool for Mycoplasma pneumoniae
Source: Front Microbiol. 2026 Oct 7;17:1979614. doi: 10.3389/fmicb.2026.1979614 (PMC13683612; doi:10.3389/fmicb.2026.1979614)
Supplement: Supplementary file 2 [file Table_2.DOCX]

Table S2. Resistance genes detection in mRT-PCR.

|  | **Strain** | **Antibiotic resistance rate of strains** |
| --- | --- | --- |
| MecA | *S. aureus* (n=2) | Oxacillin (100%) |
| VIM | *P. aeruginosa* (n=1) | Ceftazidime (100%) |
| KPC | *K. pneumoniae* (n=3), *E. coli* (n=1) | Imipenem (100%) |
| NDM | *E. coli* (n=3) | Imipenem (100%) |
| IMP | *K. pneumoniae* (n=3) | Imipenem (100%) |
| * The ampC, MCR-1, and VanA genes were not detected in this study. | | |

Data are derived from the 371-patient cohort; detections are based on mRT-PCR results for the resistance genes included in the panel.
