# Supplementary material for: Clinical validation of a novel multiplex real-time PCR for diagnosing community-acquired pneumonia in the emergency department: a promising diagnostic tool for Mycoplasma pneumoniae
Source: Front Microbiol. 2026 Oct 7;17:1979614. doi: 10.3389/fmicb.2026.1979614 (PMC13683612; doi:10.3389/fmicb.2026.1979614)
Supplement: Supplementary file 3 [file Table_3.DOCX]

**Table S3. Independent reference standard (IRS) corroboration of mRT-PCR detections in the 371-patient cohort**

The IRS excluded mRT-PCR and mNGS. For each target, an mRT-PCR positive call was counted as independently corroborated only when the identical target was identified by culture (bacteria/fungi), serum IgM (Mycoplasma pneumoniae), or routine single-plex PCR/antigen (influenza A/B, SARS-CoV-2). Targets without a routine comparator at our institution were clinically adjudicated by the physician panel (Ct value, imaging, clinical course). Sn and Sp are reported as percentages with 95% confidence intervals in parentheses; PPV and NPV are reported as percentages.

**Part A. Targets with an available independent laboratory comparator (n = 15).**

| **Target** | **Independent comparator** | **mRT-PCR calls** | **TP** | **FP** | **FN** | **TN** | **Sensitivity % (95% CI)** | **Specificity % (95% CI)** | **PPV %** | **NPV %** | **LR+** | **LR−** |
| --- | --- | --- | --- | --- | --- | --- | --- | --- | --- | --- | --- | --- |
| Streptococcus pneumoniae | Conventional sputum culture (blood & chocolate agar) | 125 | 9 | 116 | 0 | 246 | 100.0 (70.1–100.0) | 68.0 (63.0–72.6) | 7.2 | 100.0 | 3.12 | 0.00 |
| Mycoplasma pneumoniae | Serum IgM (particle agglutination) | 122 | 50 | 72 | 56 | 193 | 47.2 (37.9–56.6) | 72.8 (67.2–77.8) | 41.0 | 77.5 | 1.74 | 0.73 |
| Haemophilus influenzae | Conventional sputum culture (blood & chocolate agar) | 71 | 24 | 47 | 13 | 287 | 64.9 (48.8–78.2) | 85.9 (81.8–89.3) | 33.8 | 95.7 | 4.61 | 0.41 |
| Klebsiella pneumoniae | Conventional sputum culture (blood & chocolate agar) | 60 | 17 | 43 | 1 | 310 | 94.4 (74.2–99.0) | 87.8 (84.0–90.8) | 28.3 | 99.7 | 7.75 | 0.06 |
| Candida albicans | Conventional sputum culture (blood & chocolate agar) | 60 | 25 | 35 | 6 | 305 | 80.6 (63.7–90.8) | 89.7 (86.0–92.5) | 41.7 | 98.1 | 7.83 | 0.22 |
| Staphylococcus aureus | Conventional sputum culture (blood & chocolate agar) | 46 | 6 | 40 | 0 | 325 | 100.0 (61.0–100.0) | 89.0 (85.4–91.8) | 13.0 | 100.0 | 9.12 | 0.00 |
| SARS-CoV-2 | Routine single-plex real-time PCR / antigen | 41 | 22 | 19 | 6 | 324 | 78.6 (60.5–89.8) | 94.5 (91.5–96.4) | 53.7 | 98.2 | 14.18 | 0.23 |
| Influenza A virus | Routine single-plex real-time PCR / antigen | 35 | 25 | 10 | 3 | 333 | 89.3 (72.8–96.3) | 97.1 (94.7–98.4) | 71.4 | 99.1 | 30.63 | 0.11 |
| Pseudomonas aeruginosa | Conventional sputum culture (blood & chocolate agar) | 16 | 11 | 5 | 0 | 355 | 100.0 (74.1–100.0) | 98.6 (96.8–99.4) | 68.8 | 100.0 | 72.00 | 0.00 |
| Moraxella catarrhalis | Conventional sputum culture (blood & chocolate agar) | 15 | 2 | 13 | 0 | 356 | 100.0 (34.2–100.0) | 96.5 (94.1–97.9) | 13.3 | 100.0 | 28.38 | 0.00 |
| Acinetobacter baumannii | Conventional sputum culture (blood & chocolate agar) | 14 | 8 | 6 | 2 | 355 | 80.0 (49.0–94.3) | 98.3 (96.4–99.2) | 57.1 | 99.4 | 48.13 | 0.20 |
| Influenza B virus | Routine single-plex real-time PCR / antigen | 9 | 5 | 4 | 0 | 362 | 100.0 (56.6–100.0) | 98.9 (97.2–99.6) | 55.6 | 100.0 | 91.50 | 0.00 |
| Escherichia coli | Conventional sputum culture (blood & chocolate agar) | 6 | 1 | 5 | 0 | 365 | 100.0 (20.7–100.0) | 98.6 (96.9–99.4) | 16.7 | 100.0 | 74.00 | 0.00 |
| Aspergillus fumigatus | Conventional sputum culture (blood & chocolate agar) | 5 | 2 | 3 | 0 | 366 | 100.0 (34.2–100.0) | 99.2 (97.6–99.7) | 40.0 | 100.0 | 123.00 | 0.00 |
| Stenotrophomonas maltophilia | Conventional sputum culture (blood & chocolate agar) | 4 | 3 | 1 | 2 | 365 | 60.0 (23.1–88.2) | 99.7 (98.5–100.0) | 75.0 | 99.5 | 219.60 | 0.40 |
| Pooled (15 targets) | Summed target-specific comparisons | 629 | 210 | 419 | 89 | 4847 | 70.2 (64.8–75.1) | 92.0 (91.3–92.7) | 33.4 | 98.2 | 8.83 | 0.32 |

Pooled row: estimates across the 15 Part A targets were obtained by summing the corresponding cells of the target-specific 2x2 tables (15 x 371 = 5,565 target-patient observations). LR+ = Sn/(100 - Sp) and LR− = (100 - Sn)/Sp. Because each patient can contribute to more than one target-specific comparison, the confidence intervals shown do not adjust for within-patient clustering and should be regarded as approximate; per-target likelihood ratios for targets with few positive calls are correspondingly unstable.

**Part B. Targets without a routine comparator at our institution (clinically adjudicated).**

| **Target** | **mRT-PCR calls** | **Independently corroborated** | **Note** |
| --- | --- | --- | --- |
| Epstein-Barr virus | 85 | 0 | No routine diagnostic test available; clinically adjudicated |
| Human rhinovirus | 24 | 0 | No routine diagnostic test available; clinically adjudicated |
| Herpes simplex virus 1 | 22 | 0 | No routine diagnostic test available; clinically adjudicated |
| Human adenovirus | 20 | 0 | No routine diagnostic test available; clinically adjudicated |
| Human metapneumovirus | 15 | 0 | No routine diagnostic test available; clinically adjudicated |
| Respiratory syncytial virus | 14 | 0 | No routine diagnostic test available; clinically adjudicated |
| Cytomegalovirus | 12 | 0 | No routine diagnostic test available; clinically adjudicated |
| Chlamydia psittaci | 9 | 0 | No routine diagnostic test available; clinically adjudicated |
| Parainfluenza virus | 8 | 0 | No routine diagnostic test available; clinically adjudicated |
| Seasonal human coronavirus | 7 | 0 | No routine diagnostic test available; clinically adjudicated |
| Pneumocystis jirovecii | 6 | 0 | No routine diagnostic test available; clinically adjudicated |
| Chlamydia pneumoniae | 6 | 0 | No routine diagnostic test available; clinically adjudicated |
| Candida parapsilosis | 4 | 0 | No routine diagnostic test available; clinically adjudicated |
| Legionella pneumophila | 3 | 0 | No routine diagnostic test available; clinically adjudicated |
| Aspergillus flavus | 2 | 0 | No routine diagnostic test available; clinically adjudicated |
| Enterococcus faecium | 2 | 0 | No routine diagnostic test available; clinically adjudicated |
| Candida tropicalis | 2 | 0 | No routine diagnostic test available; clinically adjudicated |
| Aspergillus nidulans | 1 | 0 | No routine diagnostic test available; clinically adjudicated |
| Serratia marcescens | 1 | 0 | No routine diagnostic test available; clinically adjudicated |
| Enterococcus faecalis | 1 | 0 | No routine diagnostic test available; clinically adjudicated |

Note: For Part A, TP/FN are culture-, PCR-, or serology-positive calls; FP are mRT-PCR-positive calls not confirmed by the independent comparator. FP commonly reflect limitations of the comparator rather than mRT-PCR false positivity—for example, the low sensitivity of sputum culture for Streptococcus pneumoniae (especially after antibiotic exposure), the window period of Mycoplasma pneumoniae serum IgM, or the lower sensitivity of antigen tests versus nucleic-acid amplification for influenza A/B and SARS-CoV-2. TN are patients negative by both. For Part B, no routine diagnostic test exists at our institution for these targets, so independently corroborated = 0; these calls were clinically adjudicated by the physician panel using Ct value, imaging, and clinical history, and absence of a comparator is not evidence of a false positive.
